# Exome Sequencing of a Portuguese Cohort of Frontotemporal Dementia Patients: Looking Into the ALS-FTD Continuum

**DOI:** 10.3389/fneur.2022.886379

**Published:** 2022-07-07

**Authors:** Miguel Tábuas-Pereira, Isabel Santana, Elizabeth Gibbons, Kimberly Paquette, Maria Rosário Almeida, Inês Baldeiras, Jose Bras, Rita Guerreiro

**Affiliations:** ^1^Neurology Department, Centro Hospitalar e Universitário de Coimbra, Coimbra, Portugal; ^2^Faculty of Medicine, University of Coimbra, Coimbra, Portugal; ^3^Department of Innovative Biomedicine and Biotechnology (CIBB), University of Coimbra, Coimbra, Portugal; ^4^Department of Neuroscience and Cell Biology (CNC), University of Coimbra, Coimbra, Portugal; ^5^Department of Neurodegenerative Science, Van Andel Institute, Grand Rapids, MI, United States; ^6^Division of Psychiatry and Behavioral Medicine, Michigan State University College of Human Medicine, Grand Rapids, MI, United States

**Keywords:** frontotemporal dementia, ALS (amyotrophic lateral sclerosis), phenotype, cohort, Portuguese

## Abstract

**Introduction:**

Frontotemporal dementia (FTD) is considered to be part of a continuum with amyotrophic lateral sclerosis (ALS). Many genes are associated with both ALS and FTD. Yet, many genes associated with ALS have not been shown to cause FTD. We aimed to study a Portuguese cohort of FTD patients, searching for variants in genes associated with both FTD and/or ALS.

**Methods:**

We included 57 thoroughly characterized index FTD patients from our memory clinic, who were not carriers of pathogenic variants in *GRN, MAPT* or *C9orf72*. We performed exome sequencing and 1) prioritized potential FTD and ALS causing variants by using Exomiser to annotate and filter results; and 2) looked specifically at rare variability in genes associated with FTD (excluding *GRN, MAPT* and *C9ORF72*) and/or ALS.

**Results:**

We identified 13 rare missense variants in 10 patients (three patients had two variants) in the following genes: *FUS, OPTN, CCNF, DCTN1, TREM2, ERBB4, ANG, CHRNA4, CHRNB4* and *SETX*. We found an additional frameshift variant on *GLT8D1* in one patient. *One* variant (*ERBB4* p.Arg1112His) gathered enough evidence to be classified as likely pathogenic by the ACMG criteria.

**Discussion:**

We report, for the first time, an expanded study of genes known to cause FTD-ALS, in the Portuguese population. Potentially pathogenic variants in *ERBB4, FUS, SETX, ANG, CHRNA4* and *CHRNB4* were identified in FTD patients. These findings provide additional evidence for the potential role of rare variability in ALS-associated genes in FTD, expanding the genetic spectrum between the two diseases.

## Introduction

Frontotemporal dementia (FTD) is a clinically, genetically, and pathologically heterogeneous group of disorders that lead to progressive cognitive impairment due to degeneration of the frontal and temporal lobes as a common hallmark. FTD is the second most common cause of early-onset degenerative dementia (1) and about a third of its cases are found to be genetic (2). There are different genes shown to cause this disease, with most of the cases being associated with changes in three genes: progranulin (*GRN*) (3, 4), microtubule associated protein tau (*MAPT*) (5) and chromosome 9 open reading frame 72 (*C9ORF72*) (6, 7). However, the genetic landscape is rapidly expanding, with more and more genes being associated with FTD.

Over time, FTD has evolved to be considered to be part of a continuum with amyotrophic lateral sclerosis (ALS) (8). Much of the support for this association comes from genetic data, as they share many genes as the underlying cause of disease (9), such as *TARDBP, FUS, VCP, TBK1, CHCHD10, SQSTM1, UBQLN2, CCNF, CHMP2B, OPTN, DCTN1, TUBA4A, hnRNP2B1* and *hnRNPA1* (10) and most importantly, the *C9ORF72* repeat expansion (11). Yet, many of the genes that have been associated with ALS have not been shown to cause FTD (11).

In this study, we analyzed a thoroughly characterized Portuguese cohort of 57 patients with FTD. The previous screening of FTD-causative genes (*MAPT, GRN* and *C9ORF72*) allowed us to include only cases without mutations in these genes. Here we hypothesize that, given the known genetic and clinical spectrum between FTD and ALS, genes previously only related to ALS or to FTD/ALS may carry rare variants with a role in pure FTD. In this study we search for likely pathogenic variants in genes causative of both FTD (beyond *MAPT, GRN* and *C9ORF72*), and/or ALS, through exome sequencing.

## Methods

### Subjects

In this study, we included 57 FTD index patients from the memory clinic, in Centro Hospitalar e Universitário de Coimbra, Portugal, a tertiary reference hospital from the central region of Portugal. The diagnosis was performed according to the most widely accepted criteria (12, 13), and supported by extensive characterization. Our clinical protocol included (1) a complete and systematic clinical and neuropsychological evaluation; (2) structural neuroimaging [computed tomography and/or magnetic resonance imaging]; (3) lumbar puncture to determine Alzheimer's Disease (AD) cerebrospinal fluid biomarkers and/or amyloid PET, to strengthen the diagnosis, by excluding AD.

Patients are from a convenience sample recruited between 2012 and 2016. We included 57 patients, 31 of which were female (53.4%). Mean age of onset was 54.5 (34 to 73) years. All patients were from Portuguese ancestry. Of the whole cohort, twenty patients (34.4%) had positive family history (a first-degree relative with a neurodegenerative condition).

The study was approved by the ethics committee of Coimbra University Hospital and biological samples were obtained following written informed from the patients and/or the patients legal representatives.

### Exome Sequencing

Exome sequencing was performed for all patients on a HiSeq2500 with 75-100 bp pair-end reads after library preparation with the SureSelect Exome Capture Kit v4 (Agilent). Exome data processing was done following GenomeAnalysisTK best practices. Alignment was done with the Burrows-Wheeler Aligner (bwa-mem) v0.7.12 against the hg19 genome assembly. Duplicates were identified with samblaster v0.1.21 and bases were recalibrated with GenomeAnalysisTK v3.8-1 (14). Variant Quality Score Recalibration (15), and annotation with snpEff v4.2 and dbNSFP v2.9 were applied to all variants (16, 17). Variants were filtered according to their quality metrics as described by Patel and collaborators (18).

We explored variants in genes reported to cause FTD (10): *FUS, TBK1, TARDBP, SQSTM1, CHMP2B, VCP, CHCHD10, UBQLN2, CCNF, OPTN, DCTN1*, and *TUBA4A*. We also explored other genes previously linked to FTD: *TREM2, TYROBP, hnRNPA1, hnRNPA2B1* and *TMEM106B*. We further analyzed variants in genes reported to cause ALS (19), namely: *SOD1, ANG, MATR3, FIG4, VAPB, PFN1, SETX, ERBB4, ANXA11, KIF5A, PRPH, GLT8D1, TIA1, DAO, ELP3, TAF15, EWSR1, SS18L1, NEK1, C21orf2, NEFH, CHRNA3, CHRNA4, CHRNB4, PON1, PON2, and PON3*. Genes known to cause ALS in an autosomal recessive pattern and genes associated with copy number variations were not examined. All patients were previously screened for C9ORF72 expansions, GRN and MAPT mutations, and were found to be negative. Variants identified in *CYLD* in this cohort have been reported elsewhere (20).

Only rare variants were included in the study. Variants with a minor allele frequency (MAF) > 0.001 in gnomAD overall sample, or in any of the detailed subpopulations were excluded. *In silico* pathogenicity prediction was assessed using SIFT (Scale-Invariant Feature Transform) (21), Polyphen-2 (22), PROVEAN (Protein Variation Effect Analyzer) (23), Mutation Taster (24) and CADD (Combined Annotation Dependent Depletion) (25). Scores and cut-offs suggested by the software's authors were considered. In CADD, a score higher than 15 was considered as deleterious. The significant findings were comprehensively assessed by considering MAF, predicted pathogenicity, disease association, and family history.

### Exomiser Analysis

An adapted version of the Exomiser software (v12.1.0) was used to identify variants that could potentially cause the disease in the individual FTD cases by prioritizing variants related to FTD (HP:0002145) and ALS (HP: 0007354), using an autosomal dominant inheritance model.

The results from the individual runs of Exomiser analyses were filtered by excluding variants without alternative alleles, not passing the Exomiser filter (inheritance), synonymous, with low coverage, and high frequency. Variants that had heterozygous calls in more than three controls and more than 100 cases were filtered out. Variants that were not present in the available databases were kept and were sorted by the Exomiser Gene Combined score in a descending order. A list was compiled with the three top ranked candidate variants separately for FTD and ALS.

### InterVar ACMG Criteria

An adapted version of the InterVar software (v2.2.1) was used to classify variants based on ACMG guidelines (26). Out of the 28 ACMG criteria, InterVar can automatically apply 18 criteria. The variants in Table 1 were analyzed using the InterVar classification followed by a manual analysis to determine if any of the ten manual criteria were present.

**Table 1:** Rare variants identified in genes previously associated with Frontotemporal and/or Amyotrophic Lateral Sclerosis in the Portuguese cohort of Frontotemporal patients.

**Gene**	**Genomic position (hg19)**	**Zigosity**	**Variant**	**Patient**	**rs**	gnomAD MAF*	**CADD**	**PROVEAN**	**SIFT**	**Polyphen2**	**Mutation Taster**	**Additional variant**	**ACMG criteria**	**ACMG classification**
*OPTN*	10:13152343ª > G	Het	p.Gln79Arg	Patient 5	-	0.000	24.7	−2.045 (N)	0.007	0.993 (D)	0.996821 (PD)		PM2 + PP3	Unc Sig
*CCNF*	16:2487156G > A	Het	p.Glu125Lys	Patient 57	-	0.000	23.3	−1.035 (N)	0.010 (P)	0.554 (PD)	1.000(P)		PM2 + PP3	Unc Sig
*DCTN1*	2:74597375G > A	Het	p.Arg409Trp	Patient 51	rs150368544	0.00001761	27.5	−4.761 (D)	0.0 (P)	0.913 (D)	0.99239 (P)		PM1 + PM2 + PP3	Unc Sig
*FUS*	16:31201719C > T	Het	p.Pro432Leu	Patient 35	rs773104641	0.0001497	7.163	−5.95 (D)	0.001 (D)	1.000 (D)	1.000 (P)	*SETX* p.Thr372Pro	PM1 + PM2 + PP3	Unc Sig
*TREM2*	6:41129275G > C	Het	p.Asp39Glu	Patient 10	rs200392967	0.0001259	23.8	−1.254 (N)	0.13(T)	0.246 (B)	0.91608 (Pm)		PM2	Unc Sig
*ERBB4*	2:212251724C > T	Het	p.Arg1112His	Patient 23	rs770460785	0.00002324	23.0	−1.566 (N)	0.032	0.003 (B)	1.000 (P)	*CHRNB4* p.Val220Met	PM1 + PP2 + PP2 + PP3	Likely Pathogenic
*ANG*	14:21162091G > C	Het	p.Gly123Ala	Patient 32	-	0.00006193	0.06	−1.233 (N)	0.1	0.048 (B)	1.000 (Pm)		PM1 + PM2 + BS4	Unc Sig
*SETX*	9:135205871T > G	Het	p.Thr372Pro	Patient 34	rs145145045	0.00002328	22.4	−0.962 (N)	0.002	0.073	0.99786 (Pm)	FUS p.Pro431Leu	PM2	Unc Sig
*SETX*	9:135202313T > C	Het	p.Thr1558Ala	Patient 16	rs764920626	0.000008822	0.002	−0.233 (N)	0.243	0.015	1.000 (Pm)	*CHRNA4* p.Thr545Met	PM2 + BP4	Unc Sig
*SETX*	9:135211763G > A	Het	p.Ser213Phe	Patient 13	rs1254442456	0.000	28.1	−2.093 (N)	0.0	0.998 (D)	0.87885 (P)		PM2 + PP3	Unc Sig
*CHRNA4*	20:61981129G > A	Het	p.Thr545Met	Patient 16	rs121912282	0.00007112	2.412	−0.01 (N)	0.124 (T)	0.862 (D)	1.000 (Pm)	*SETX* p.Thr1558Ala	PM1 + PM2 + BP4	Unc Sig
*CHRNB4*	15:78927807G > A	Het	p.Leu60Phe	Patient 29	-	0.000	25.1	−2.95 (D)	0.001 (D)	0.726 (D)	0.82638 (P)		PM1 + PM2 + PP3	Unc Sig
*CHRNB4*	15:78921989C > T	Het	p.Val220Met	Patient 23	rs774714066	0.0001161	20.6	−0.62 (N)	0.101 (T)	0.999 (D)	0.9995 (P)	*ERBB4 p.Arg1112His*	PM1 + PM2 + PP3	Unc Sig
*GLT8D1*	3:52730611 GT > G	Het	p.Lys131fs	Patient 41	rs1308367710	0.000		NA	NA				PM2 + PM4 + BS4	Unc Sig

^*^gnomAD MAFs correspond to non-Finnish Europeans. Pt, Patient; Het, heterozygous; rs, reference SNP; MAF, Minor Allele Frequency; ACMG, American College of Medical Genetics (27); CADD, Combined Annotation Dependent Depletion; PROVEAN, Protein Variation Effect analyzer; SIFT, Scale-Invariant Feature Transform; Unc Sig(28), Unclear Significance; PM, Pathogenic Moderate [number according to ACMG guidelines (27)]; PP, Pathogenic Supporting [number according to ACMG guidelines (27)]; BP, Benign Supporting [number according to ACMG guidelines (27)]; N: Neutral; D: Damaging, T: Tolerable; P: Pathogenic; B: Benign; PD: Probably Damaging; Pm: Polymorphism.

## Results

We identified 14 rare variants (13 missense and one frameshift variant) in 11 patients (three patients had two variants), which are reported in Table 1.

We found rare missense variants in *FUS, OPTN, DCTN1, ERBB4, ANG, TREM2, CCNF, CHRNA4, CHRNB4* and *SETX* and a frameshift variant in *GLT8D1* (p.Lys131fs^*^7), in one patient.

For several of the analyzed genes we did not identify any relevant variants: *TBK1, TARDBP, VCP, TUBA4A, UBQLN2, SQSTM1, CHMP2B, CHCHD10, hnRNPA1, hnRNPA2B1, TYROBP* and *TMEM106B*. This was also true for the ALS genes *SOD1, PFN1, VAPB, MATR3, ELP3, TAF15, EWSR1, FIG4, SIGMAR1, NEFH, DAO, KIF5A, TIA1, C21orf2, NEK1, SS18L1, ANXA11, CHRNA3, PRPH, PON1, PON2, and PON3*.

The top three results from the Exomiser analysis for each FTD case are presented in Supplementary Table 1. None of the variants identified were a clear cause of disease in the respective cases. Some of the top results were identified in the same genes studied here, including *MATR3, TREM2, SQSTM1*, and *DCTN1*.

## Discussion

Frontotemporal dementia is thought to be part of a spectrum with ALS (8). Although only 10% of ALS cases are considered to be familial (28), the heritability of ALS sums up to 60% (27). Currently, over 25 genes have been associated with ALS (29), but only a fraction of these has also been implicated in FTD. FTD has a strong genetic component but in many familial cases a genetic cause remains to be identified. One may speculate that some of those cases can be attributed to genes which have, so far, been described in ALS cases only.

We studied a sample of 57 FTD Portuguese patients, by performing exome sequencing and (1) prioritizing potential FTD and ALS causing variants by using Exomiser to annotate and filter results; and (2) looking specifically at rare variability in genes associated with FTD (excluding *GRN, MAPT* and *C9ORF72*) and/or ALS.

No clearly pathogenic variants were identified in the top results from Exomiser, but some of the prioritized genes overlapped with the genes studied here and known to have a role in FTD and/or ALS. Noteworthy, there was one *GRN* variant (p.Gly70Ser in patient 5), which has been found in controls (30) and one *APP* variant (p.Ala500Thr in patient 18), which has also been found in controls (31).

We identified rare variants in *FUS, OPTN, CCNF* and *DCTN1*, adding support to the role of these genes in FTD. Regarding genes previously only associated with ALS, we identified rare variants in *ERBB4, ANG, CHRNA4, CHRNB4, SETX* and *GLT8D1*, some of them predicted to be pathogenic *in silico*. These findings suggest that these ALS genes may also have a role in the pathogenesis of FTD. The absence of significant results in the FTD genes studied here may reflect the strong role of *GRN, MAPT* and *C9ORF72* in the disease. Cases with variants predicted to be pathogenic in these genes were excluded from the cohort to increase the probability of finding new genetic factors associated with the disease.

When comparing the phenotypic features between patients harboring rare genetic variants in the ALS genes with those without such variants, we did not see any specific phenotypic differences. However, given the small size of our cohort and the fact that we are considering different genes, such a pattern would be difficult to notice.

### Genes Previously Associated With FTD

We identified a novel *OPTN* variant (p.Gln79Arg), predicted to be pathogenic *in silico*, in a male patient with progressive primary aphasia, starting at 62 years old (patient 5). This variant is absent from gnomAD, has a high CADD score (24.7), and leads to a change in the NEMO-like domain of the protein, thought to interact with *TBK1* (32). So far most variants reported in ALS are located at the end of this domain (p.Ala93Pro, p.Lys95Asn, p.Arg96Leu), but there are some variants, namely p.Glu50Lys, which have been shown to be pathogenic, causing normal tension glaucoma (32).

Another variant absent from gnomAD was identified in *CCNF* (p.Glu125Lys) in a female patient diagnosed with bvFTD at age 48 (patient 57). *CCNF* encodes the Cyclin F protein that has been shown to cause abnormal ubiquination and accumulation of ubiquinated proteins, including TDP-43 when mutated (33). This variant is predicted to be pathogenic by SIFT, Polyphen 2 and Mutation Taster and is located between the F-box and the cyclin D regions of the protein (33). Other variants have been described in this region, such as p.Lys97Arg, p.Thr181Ile and p.Ser195Arg (33). These variants have been shown to cause the mislocalization of Cyclin F to the cytoplasm and increased association of Cyclin F with VCP, resulting in activation of VCP ATPase, which plays a positive role in TDP-43 aggregation (34).

For both variants, in *OPTN* and *CCNF* the *in silico* analyses performed pointed to potential deleterious effects. In addition, the absence of these variants from gnomAD supports a potential pathogenic role in disease. Still, these variants had uncertain significance when using the ACMG guidelines and further evidence would need to be found to support their pathogenicity.

Other rare variants were identified in *DCTN1, FUS* and *TREM2*, but contrary to the ones described above, these are all reported in gnomAD.

The *DCTN1* variant (p.Arg409Trp), although rare and predicted to be pathogenic *in silico*, is located outside the cytoskeleton-associated protein-glycine-rich domain of the protein, where all the variants reported to date and considered to be pathogenic are located (35). This was found in another female patient with bvFTD, starting at the age of 48 (patient 51). This variant was also determined to be of uncertain significance by the ACMG guidelines and further evidence would be needed to support its pathogenicity.

*FUS* mutations in pure FTD are rare (36). Here, one female bvFTD patient with an age at onset of 53 years (patient 34) carried a missense variant (Pro432Leu), predicted to be pathogenic *in silico*. This variant has been previously associated with familial essential tremor (37). The proline at position 432 is highly conserved and located in the zinc finger domain. It may affect splicing as it affects the last nucleotide of exon 12. However, no other variants in this domain have been associated with ALS or FTD (36) and this was found to be of uncertain significance by the ACMG guidelines. This patient also carried a rare *SETX* variant (Table 1).

In *TREM2*, we identified an heterozygous missense variant (p.Asp39Glu), previously described and of unclear pathogenicity in FTD (38). This variant was found in a female patient with a primary progressive aphasia variant, starting at the age of 61 (patient 10).

### Variants in Genes Associated Only With ALS

One of the main aims of this study was to search for rare, potentially pathogenic, variants in genes associated with ALS with no previous association with FTD. Specifically looking into these genes, we identified rare variants in *ERBB4, ANG, SETX, CHRNA4, CHRNB4* and *GLT8D1*, the latter being a frameshift variant present in one patient.

Recently, a variant in *ERBB4* has been described in one FTD-ALS patient (39), supporting the idea that genes associated with ALS may be also implicated in FTD pathogenesis. However, the assessment of cognitive impairment in that patient was complicated by dysarthria and no clear frontal impairment was documented in the study. We report a variant (p.Arg1112His, in patient 23), which is predicted to be pathogenic *in silico* by SIFT and Mutation Taster. This, together with the previously described ALS-FTD case, gives some support to the potential for this gene to be involved in FTD. This variant was classified as likely pathogenic by the ACMG guidelines.

Angiogenin (*ANG*) is a pro-angiogenic and neurotrophic factor with an important role in stress-induced injury, that acts by promoting neovascularization and neuronal survival (40). Pathogenic variants in this gene have been implicated in ALS (41). Interestingly, in an ALS-FTD cohort, the levels of angiogenin in CSF were only elevated in patients with FTD (40) and at least one patient reported in the literature seemed to have ALS-FTD (42). The variant we identified (p.Gly123Ala) is located close to the previously reported variants p.Arg121His and p.Arg122His (43, 44). The patient carrying this variant is part of a family with 3 of 4 siblings affected, and this variant was only present in two of them. It was also present in one descendant, currently not affected. This, together with the *in silico* data, argues against the pathogenicity of this variant, or, less likely, the presence of a phenocopy in the family.

Senataxin (*SETX)* is associated with juvenile ALS in an autosomal dominant inheritance pattern and with ataxia with oculomotor apraxia type 2 in a recessive inheritance pattern. Senataxin is a protein associated with R-loop regulation. An R-loop forms when an RNA transcript displaces homologous DNA sequence of one of the DNA strands of chromosomes and hybridizes to the complementary sequence of the other strand, generating a short region of RNA-DNA hybrid and single stranded DNA. R-loop dysfunction is increasingly recognized as the cause of several disorders, such as *C9orf72* associated ALS (45). In our cohort, we identified three rare *SETX* variants. One patient carried a variant (p.Thr1558Ala, in patient 16) close to a previously reported pathogenic variant (p.Thr1863Ala) (46). Other rare variant identified in this Portuguese cohort was p.Thr372Pro (patient 34), located in the helicase domain. The third variant was p.Ser213Phe, the only one predicted to be deleterious. This variant is also not reported in gnomAD. This variant was found in a male patient with bvFTD starting at the age of 55 (patient 13).

*CHRNA3, CHRNA4* and *CHRNB4* have been associated with ALS (47). These genes encode neuronal nicotinic acetylcholine receptors and regulate Ca2^+^ influx to the neurons. Rare missense variants in the intracellular domains of these proteins have been shown to be more prevalent in sporadic ALS patients (48). They also have been shown to be associated with cognitive performance (49). In particular, *CHRNB4* has been linked to the modulation of attention in attention deficit/hyperactivity disorder (50). Together, these data point to the possible role of variability in FTD risk and/or pathogenesis. We identified three rare variants in this gene cluster, with two variants located in *CHRNB4* (p.Leu60Phe, in patient 29, and p.Val220Met, in patient 23) being particularly relevant, as they are very rare or absent in gnomAD and predicted to be pathogenic *in silico* by five of five (p.Leu60Phe) and three of five (p.Val220Met) tools (Table 1). However, all three of these variants were classified with uncertain significance by the ACMG guidelines.

Finally, we found one female patient (patient 42) with an age at onset of FTD at 66 years carrying a frameshift variant in *GLT8D1*, a gene recently associated with ALS (51). It encodes a glycosyltransferase enzyme of unknown function, possibly linked to ganglioside synthesis. *GLT8D1* has also been linked with schizophrenia (52, 53). So far, the vast majority of variants reported to cause ALS are missense and located in exon 4 (51). One variant in exon 10 has also been described in a Chinese cohort (47). The variant identified in our cohort (which is not present in gnomAD) leads to a change in the same glycosyltransferase domain of the protein but is predicted to cause a premature stop codon. It is unclear if variants causing a truncated protein will have the same functional effect as the missense variants previously identified in ALS. However, given that in this case we were able to assess the sister of the proband that also presented with FTD-ALS and did not carry the variant, the likelihood of pathogenicity for this variant is very low.

This study has some limitations, including the small size of the cohort, the lack of pathological diagnoses and the unavailability of samples from family members to test co-segregation of variants with disease. Only one of the FTD patients had a pathological confirmation of diagnosis, although all patients in the cohort were clinically evaluated and thoroughly studied in an experienced dementia focused centre. Due to the absence of family history or the unavailability of samples from family members we did not perform segregation analyses to further study the pathogenicity of most of the reported variants. However, the frequency of the variants, together with the use of *in silico* prediction tools and functional annotation allowed us to weight the possible role of the identified variants.

This study described for the first time the genetic variability beyond *GRN, MAPT* and *C9ORF72*, in a clinically well characterized cohort of FTD cases from Portugal. The identification of rare variants in *FUS, SETX, CHRNB4, CHRNA4* and *ERBB4* in FTD patients provides additional support for a role of ALS-associated genes in pure FTD cases. This expands the spectrum between FTD and ALS on a genetic level. Replication and extension of these findings can have a significant impact on the genetic counseling of affected patients and families and on the understanding of FTD mechanisms.

## Data Availability Statement

The variants identified in this study have been submitted into online repositories. The name of the repository and accession numbers can be found below: National Center for Biotechnology Information (NCBI) ClinVar, https://www.ncbi.nlm.nih.gov/clinvar/, VCV001344510-VCV001344518, VCV000586548, VCV000648503, VCV000905893, VCV000037069, and VCV000098320.

## Ethics Statement

The studies involving human participants were reviewed and approved by Ethics Committee of Coimbra University Hospital, Coimbra, Portugal. The patients/participants provided their written informed consent to participate in this study.

## Author Contributions

MT-P has drafted the manuscript and prepared the final version. MT-P, RG, and JB have designed the study. IS, MT-P, IB, and MA have contributed in the collection of samples and patients study. MT-P, EG, KP, MA, RG, and JB have contributed to the analysis of the bioinformatic data. MT-P, IS, KP, EG, RG, and JB have reviewed the manuscript. All authors contributed to the article and approved the submitted version.

## Funding

Research reported in this publication was supported by the National Institute on Aging of the National Institutes of Health under Award Number R01AG067426.

## Author Disclaimer

The content is solely the responsibility of the authors and does not necessarily represent the official views of the National Institutes of Health.

## Conflict of Interest

The authors declare that the research was conducted in the absence of any commercial or financial relationships that could be construed as a potential conflict of interest.

## Publisher's Note

All claims expressed in this article are solely those of the authors and do not necessarily represent those of their affiliated organizations, or those of the publisher, the editors and the reviewers. Any product that may be evaluated in this article, or claim that may be made by its manufacturer, is not guaranteed or endorsed by the publisher.
