# Supplementary material for: Exome Sequencing of a Portuguese Cohort of Frontotemporal Dementia Patients: Looking Into the ALS-FTD Continuum
Source: Front Neurol. 2022 Jul 7;13:886379. doi: 10.3389/fneur.2022.886379 (PMC9300853; doi:10.3389/fneur.2022.886379)
Supplement: Supplementary file 1 [file Table_1.DOCX]

**Supplementary Material**

**Exome sequencing of a Portuguese cohort of Frontotemporal dementia patients: looking into the ALS-FTD continuum**

Miguel Tábuas-Pereira, Isabel Santana, Elizabeth Gibbons, Kimberly Paquette, Maria Rosário Almeida, Inês Baldeiras, José Brás, Rita Guerreiro

**Results**

Supplementary Table 1. Top three variants and genes for each patient from FTD Exomiser analysis and ALS Exomiser analysis.

| **Sample** | **Chr** | **Pos** | **Ref** | **Alt** | **Zygosity** | **Variant type** | **Gene** | **Exomiser score** | **Rsid** | **Variant (protein)** | **Variant (DNA)** |
| --- | --- | --- | --- | --- | --- | --- | --- | --- | --- | --- | --- |
| Patient 1 | chr16 | 2496827 | C | T | Het | missense | TBC1D24 | 0.9512829 | rs748302886 | p.Arg227Trp | c.679C>T |
|  | chr2 | 73641278 | C | G | Het | missense | NAT8 | 0.7853287 | . | p.Lys117Asn | c.351G>C |
|  | chr17 | 17817781 | G | C | Het | missense | SREBF1 | 0.7836751 | rs149397348 | p.Pro470Arg | c.1409C>G |
|  | chr4 | 127832704 | C | A | Het | missense | HSPA4L | 0.7634619 | rs1196985707 | p.Pro784Thr | c.2350C>A |
|  | chr19 | 288147 | G | A | Het | missense | PLPP2 | 0.75858766 | rs376789309 | p.Thr47Met | c.140C>T |
|  | chr17 | 10443439 | G | T | Het | missense | MYH4 | 0.75736815 | rs200121757 | p.Ser1919Tyr | c.5756C>A |
| Patient 2 | chr3 | 38739639 | A | G | Het | missense | SCN10A | 0.7625183 | rs758843257 | p.Phe719Ser | c.2156T>C |
|  | chr16 | 2088564 | G | A | Het | missense | TSC2 | 0.7614203 | rs45506695 | p.Arg1793Gln | c.5378G>A |
|  | ***chr 15** | **63279326** | **CTT** | **C** | **Het** | **Frameshift truncation** | **APH1B** | **0.7580891** | **.** | **p.Leu94fs** | **c.280281delTT** |
|  | chr4 | 95125075 | T | A | Het | missense | BMPR1B | 0.7930536 | rs776445882 | p.Ile210Asn | c.629T>A |
|  | ***chr 15** | **63279326** | **CTT** | **C** | **Het** | **Frameshift truncation** | **APH1B** | **0.7577926** | **.** | **p.Leu94fs** | **c.280_281delTT** |
|  | chr16 | 71658690 | C | T | Het | missense | PHLPP2 | 0.75652826 | rs776753936 | p.Ser739Asn | c.2216G>A |
| Patient 3 | chr22 | 17203770 | C | G | Het | missense | ADA2 | 0.97952706 | rs1409998636 | p.Leu182Phe | c.546G>C |
|  | chr11 | 1009353 | T | C | Het | missense | AP2A2 | 0.79279166 | . | p.Phe856Leu | c.2566T>C |
|  | chr19 | 15291825 | G | A | Het | missense | NOTCH3 | 0.78131104 | Rs143695196 | p.His981Tyr | c.2941C>T |
|  | chr6 | 36136717 | G | A | Het | missense | MAPK13 | 0.77545255 | rs376384508 | . | c.557G>A |
|  | chr15 | 64136365 | C | A | Het | missense | SNX1 | 0.76899356 | . | p.Phe467Leu | c.1401C>A |
|  | chr2 | 202883683 | T | A | Het | missense | WDR12 | 0.7531088 | rs1422996122 | p.Lys349Asn | c.1047A>T |
| Patient 4 | chr11 | 66710742 | G | A | Het | missense | SPTBN2 | 0.78999645 | rs372293906 | p.Arg305Cys | c.913C>T |
|  | chr7 | 100888933 | T | TTGCC | Het | Frameshift elongation | UFSP1 | 0.7691319 | . | p.Glu114fs | c.335338dupGGCA |
|  | chr6 | 151880688 | A | G | Het | missense | ESR1 | 0.76130277 | rs1182554992 | p.Gln226Arg | c.677A>G |
|  | chr5 | 51387746 | G | T | Het | missense | ISL1 | 0.9529578 | . | p.Ala159Ser | c.475G>T |
|  | chr5 | 149348364 | T | C | Het | missense | GRPEL2 | 0.75581264 | rs772982005 | p.Leu57Pro | c.170T>C |
|  | chr20 | 49984193 | G | A | Het | missense | SNAI1 | 0.75509506 | . | p.Arg151Gln | c.452G>A |
| Patient 5 | chr17 | 44349495 | G | A | Het | missense | GRN | 0.98588055 | rs772381732 | p.Gly70Ser | c.208G>A |
|  | ***chr 15** | **89333612** | **TGCTGCTGCTGCTGCTGCC** | **T** | **Het** | **Disruptive inframe deletion** | **POLG** | **0.9798247** | **.** | **p.Arg42Gln47del** | **c.125142delGGCAGCAGCAGCAGCAGC** |
|  | chr19 | 13299007 | C | T | Het | missense | CACNA1A | 0.9426037 | rs763054302 | p.Gly880Ser | c.2638G>A |
|  | ****chr10** | **13110343** | **A** | **G** | **Het** | **missense** | **OPTN** | **0.9980943** | **.** | **p.Gln79Arg** | **c.236A>G** |
|  | chr21 | 44312234 | A | T | Het | missense | PFKL | 0.82649827 | rs930410431 | p.Thr123Ser | c.367A>T |
|  | ***chr 15** | **89333612** | **TGCTGCTGCTGCTGCTGCC** | **T** | **Het** | **Disruptive inframe deletion** | **POLG** | **0.78877246** | **.** | **p.Arg42_Gln47del** | **c.125_142delGGCAGCAGCAGCAGCAGC** |
| Patient 6 | ***chr 11** | **121557371** | **C** | **T** | **Het** | **missense** | **SORL1** | **0.9970898** | **rs769806986** | **p.Arg877Cys** | **c.2629C>T** |
|  | chr19 | 1048921 | A | G | Het | missense | ABCA7 | 0.99350566 | . | p.Asn766Asp | c.2296A>G |
|  | chr1 | 183127356 | G | T | Het | missense | LAMC1 | 0.837737 | . | p.Trp1025Cys | c.3075G>T |
|  | ***chr 11** | **121557371** | **C** | **T** | **Het** | **missense** | **SORL1** | **0.8757996** | **rs769806986** | **p.Arg877Cys** | **c.2629C>T** |
|  | chr11 | 75449980 | C | T | Het | missense | GDPD5 | 0.8602042 | rs149542583 | p.Gly127Arg | c.379G>A |
|  | chr18 | 27985059 | C | G | Het | missense | CDH2 | 0.85644406 | rs201543789 | p.Gly717Ala | c.2150G>C |
| Patient 7 | chr11 | 73977015 | C | T | Het | missense | UCP2 | 0.9332495 | . | p.Ala114Thr | c.340G>A |
|  | chr11 | 64901976 | C | T | Het | missense | ATG2A | 0.8209641 | rs143404952 | p.Gly1369Ser | c.4105G>A |
|  | chr1 | 11846010 | C | T | Het | Splice donor | NPPA | 0.7660281 | rs755829943 | . | c.453+2G>A |
|  | chr6 | 44445815 | C | G | Het | missense | CDC5L | 0.764464 | . | p.Thr751Ser | c.2252C>G |
|  | chr20 | 1604807 | A | G | Het | missense | SIRPB1 | 0.7568434 | rs1327784969 | p.Val229Ala | c.686T>C |
|  | chr1 | 16129539 | C | T | Het | missense | EPHA2 | 0.7565971 | rs765722529 | p.Arg907His | c.2720G>A |
| Patient 8 | chr4 | 6300654 | C | G | Het | Splice region | WFS1 | 0.8831613 | rs201889635 | . | c.862-3C>G |
|  | chr14 | 96285942 | C | T | Het | missense | ATG2B | 0.8475817 | rs776040137 | p.Arg2017Gln | c.6050G>A |
|  | ***chr 12** | **57233838** | **A** | **C** | **Het** | **missense** | **SHMT2** | **0.7606738** | **rs1354527520** | **p.Ile405Leu** | **c.1213A>C** |
|  | ***chr 12** | **57233838** | **A** | **C** | **Het** | **missense** | **SHMT2** | **0.7705377** | **rs1354527520** | **p.Ile405Leu** | **c.1213A>C** |
|  | chr2 | 208288812 | G | A | Het | missense | PIKFYVE | 0.7673793 | rs187383957 | p.Arg302Gln | c.905G>A |
|  | chr16 | 2472814 | C | T | Het | missense | NTN3 | 0.76704794 | rs35489568 | p.Arg348Cys | c.1042C>T |
| Patient 9 | chr22 | 23764404 | T | C | Het | Splice donor | C22orf15 | 0.8908136 | . | . | c.255+2T>C |
|  | chr19 | 17273942 | T | C | Het | missense | BABAM1 | 0.7688035 | rs1313122615 | p.Ile128Thr | c.383T>C |
|  | ***chr 16** | **68361247** | **C** | **T** | **Het** | **missense** | **SMPD3** | **0.763813** | **.** | **p.Ala643Thr** | **c.1927G>A** |
|  | chr2 | 120982729 | C | G | Het | missense | GLI2 | 0.9565698 | rs372925840 | p.Ser511Trp | c.1532C>G |
|  | chr13 | 113164597 | C | G | Het | missense | PROZ | 0.8309053 | . | p.Ala175Gly | c.524C>G |
|  | ***chr 16** | **68361247** | **C** | **T** | **Het** | **missense** | **SMPD3** | **0.76370895** | **.** | **p.Ala643Thr** | **c.1927G>A** |
| Patient 10 | ****chr6** | **41161537** | **G** | **C** | **Het** | **missense** | **TREM2** | **0.9978232** | **rs200392967** | **p.Asp39Glu** | **c.117C>G** |
|  | chr3 | 184320694 | G | A | Het | missense | EIF4G1 | 0.9786215 | rs34838305 | p.Arg208His | c.623G>A |
|  | ***chr6** | **72399006** | **A** | **G** | **Het** | **missense** | **RIMS1** | **0.7755869** | **.** | **p.Lys1591Arg** | **c.4772A>G** |
|  | ****chr6** | **41161537** | **G** | **C** | **Het** | **missense** | **TREM2** | **0.9937489** | **rs200392967** | **p.Asp39Glu** | **c.117C>G** |
|  | ***chr6** | **72399006** | **A** | **G** | **Het** | **missense** | **RIMS1** | **0.7746677** | **.** | **p.Lys1591Arg** | **c.4772A>G** |
|  | chr10 | 100488855 | T | TA | Het | Splice donor variant | SEC31B | 0.7645432 | . | . | c.3288+2dupT |
| Patient 11 | chr16 | 70268293 | A | G | Het | missense | AARS1 | 0.9511683 | rs775444020 | p.Val350Ala | c.1049T>C |
|  | chr19 | 14100903 | G | C | Het | missense | PRKACA | 0.7683798 | . | p.Asn114Lys | c.342C>G |
|  | chr19 | 12947903 | T | C | Het | missense | RAD23A | 0.76262563 | . | p.Val43Ala | c.128T>C |
|  | chr21 | 28943686 | C | G | Het | missense | LTN1 | 0.95617145 | rs376034413 | p.Val1447Leu | c.4339G>C |
|  | chr3 | 184170177 | G | A | Het | missense | DVL3 | 0.8081161 | . | p.Gly557Asp | c.1670G>A |
|  | chr2 | 208339478 | T | G | Het | missense | PIKFYVE | 0.78963435 | . | p.Leu1578Trp | c.4733T>G |
| Patient 12 | chr3 | 132332219 | C | T | Het | missense | ACPP | 0.7900815 | rs368005227 | p.Arg111Trp | c.331C>T |
|  | chr11 | 3120569 | A | G | Het | missense | OSBPL5 | 0.78131104 | rs142011119 | p.Val153Ala | c.458T>C |
|  | chr3 | 186853222 | G | A | Het | missense | ADIPOQ | 0.76794106 | rs143606172 | p.Arg55His | c.164G>A |
|  | chr4 | 8227805 | C | T | Het | missense | SH3TC1 | 0.8830364 | rs749808678 | p.Ala704Val | c.2111C>T |
|  | chr12 | 12721119 | A | G | Het | missense | CDKN1B | 0.77012986 | rs1428339523 | p.His177Arg | c.530A>G |
|  | chr3 | 38698454 | G | T | Het | missense | SCN10A | 0.7551568 | rs753004431 | p.Ala1589Glu | c.4766C>A |
| Patient 13 | chr2 | 177728141 | G | T | Het | missense | PDE11A | 0.9457138 | rs763216873 | p.Ala607Asp | c.1820C>A |
|  | chr16 | 67285478 | G | A | Het | missense | AC040160.1 | 0.84762865 | rs199603402 | p.Arg795His | c.2384G>A |
|  | chr12 | 12183986 | T | C | Het | missense | LRP6 | 0.7889816 | rs780074436 | p.Lys324Glu | c.970A>G |
|  | ****chr9** | **132336376** | **G** | **A** | **Het** | **missense** | **SETX** | **0.9949615** | **rs1254442456** | **p.Ser213Phe** | **c.638C>T** |
|  | chr15 | 81300409 | C | T | Het | missense | IL16 | 0.83838236 | rs1208070828 | p.Ser1028Phe | c.3083C>T |
|  | chr12 | 4379495 | G | A | Het | missense | FGF23 | 0.8119534 | rs758725402 | p.Pro30Ser | c.88C>T |
| Patient 14 | chr12 | 40322047 | G | T | Het | missense | LRRK2 | 0.97937053 | rs145364431 | p.Arg1728Leu | c.5183G>T |
|  | chr1 | 59378800 | G | A | Het | missense | FGGY | 0.8549029 | . | p.Asp173Asn | c.517G>A |
|  | ***chr 18** | **28003067** | **G** | **A** | **Het** | **missense** | **CDH2** | **0.7896043** | **rs753401951** | **p.Ser317Leu** | **c.950C>T** |
|  | ***chr 18** | **28003067** | **G** | **A** | **Het** | **missense** | **CDH2** | **0.85695416** | **rs753401951** | **p.Ser317Leu** | **c.950C>T** |
|  | chr4 | 95125060 | C | G | Het | missense | BMPR1B | 0.79066354 | rs367777041 | p.Ser205Cys | c.614C>G |
|  | chr22 | 23160948 | T | C | Het | missense | RAB36 | 0.76774484 | rs764563046 | p.Leu296Pro | c.887T>C |
| Patient 15 | chr12 | 9090000 | C | A | Het | missense | A2M | 0.97615904 | rs373992205 | p.Ala874Ser | c.2620G>T |
|  | chr16 | 10895324 | GT | G | Het | Frameshift truncation | CIITA | 0.79850537 | . | p.Gly33fs | c.96delT |
|  | ***chr7** | **159030668** | **T** | **G** | **Het** | **missense** | **VIPR2** | **0.7886811** | **rs1282615864** | **p.His563Pro** | **c.1688A>C** |
|  | ***chr7** | **159030668** | **T** | **G** | **Het** | **missense** | **VIPR2** | **0.78785455** | **rs1282615864** | **p.His563Pro** | **c.1688A>C** |
|  | chr11 | 65096284 | G | A | Het | missense | VPS51 | 0.7694918 | rs757532613 | p.Gly12Arg | c.34G>A |
|  | chr15 | 65375279 | C | T | Het | missense | IGDCC3 | 0.75782686 | . | p.Arg76Lys | c.227G>A |
| Patient 16 | chr2 | 166311734 | C | G | Het | missense | SCN9A | 0.95059043 | rs747772882 | p.Gly8Ala | c.23G>C |
|  | chr14 | 100149555 | C | T | Het | missense | DEGS2 | 0.76377517 | rs1010473914 | p.Val80Met | c.238G>A |
|  | chr20 | 64205704 | G | C | Het | missense | MYT1 | 0.7606112 | rs1043869339 | p.Asp101His | c.301G>C |
|  | ****chr9** | **132326926** | **T** | **C** | **Het** | **missense** | **SETX** | **0.9571163** | **rs764920626** | **p.Thr1558Ala** | **c.4672A>G** |
|  | chr18 | 57353009 | T | C | Het | missense | ST8SIA3 | 0.8423745 | rs749514653 | p.Phe55Leu | c.163T>C |
|  | chr7 | 132133054 | C | T | Het | missense | PLXNA4 | 0.7676244 | rs771291757 | p.Glu1862Lys | c.5584G>A |
| Patient 17 | ***chr3** | **187729966** | **G** | **A** | **Het** | **missense** | **BCL6** | **0.85976404** | **rs754091486** | **p.Arg147Trp** | **c.439C>T** |
|  | chr16 | 23194193 | C | G | Het | missense | SCNN1G | 0.76913714 | rs1269700079 | p.Pro278Ala | c.832C>G |
|  | chr2 | 201407567 | A | G | Het | missense | TRAK2 | 0.76586837 | rs1367473409 | p.Val41Ala | c.122T>C |
|  | chr16 | 70324591 | G | C | Het | missense | DDX19B | 0.7686827 | . | p.Gln137His | c.411G>C |
|  | chr19 | 19316525 | G | A | Het | Stop gained | SUGP1 | 0.764441 | . | p.Gln35* | c.103C>T |
|  | ***chr3** | **187729966** | **G** | **A** | **Het** | **missense** | **BCL6** | **0.7565454** | **rs754091486** | **p.Arg147Trp** | **c.439C>T** |
| Patient 18 | chr21 | 25955716 | C | T | Het | missense | APP | 0.99710166 | rs201547994 | p.Ala500Thr | c.1498G>A |
|  | chr16 | 10908121 | G | A | Het | missense | CIITA | 0.7974046 | rs772620752 | p.Val878Met | c.2632G>A |
|  | chr13 | 24690447 | G | C | Het | missense | ATP12A | 0.7625591 | rs374324240 | p.Arg219Thr | c.656G>C |
|  | chr14 | 101986327 | A | C | Het | Missense | DYNC1H1 | 0.99107265 | . | p.Asp701Ala | c.2102A>C |
|  | chr22 | 37983605 | G | C | Het | Missense | SOX10 | 0.9601007 | . | p.Asp60Glu | c.180C>G |
|  | chr5 | 37051819 | C | T | Het | missense | NIPBL | 0.8421492 | . | p.Pro2332Leu | c.6995C>T |
|  | chr7 | 27141951 | A | G | Het | missense | HOXA5 | 0.79512364 | rs1473321001 | p.Cys233Arg | c.697T>C |
| Patient 19 | chr5 | 177385775 | G | A | Het | missense | SLC34A1 | 0.784861 | rs1047240450 | p.Ala12Thr | c.34G>A |
|  | ***chr 20** | **44930469** | **G** | **C** | **Het** | **missense** | **PABPC1L** | **0.76310086** | **.** | **p.Glu328Gln** | **c.982G>C** |
|  | chr17 | 7709538 | C | T | Het | missense | EFNB3 | 0.91827756 | rs532773147 | p.Pro329Ser | c.985C>T |
|  | chr4 | 142160532 | A | T | Het | missense | INPP4B | 0.781811 | rs546228880 | p.Asp463Glu | c.1389T>A |
|  | ***chr 20** | **44930469** | **G** | **C** | **Het** | **missense** | **PABPC1L** | **0.76543707** | **.** | **p.Glu328Gln** | **c.982G>C** |
|  | ***chr 12** | **40340373** | **G** | **A** | **Het** | **missense** | **LRRK2** | **0.97982377** | **.** | **p.Ala2010Thr** | **c.6028G>A** |
| Patient 20 | ***chr 12** | **111341933** | **A** | **G** | **Het** | **missense** | **CUX2** | **0.95166695** | **.** | **p.Lys1180Arg** | **c.3539A>G** |
|  | chr9 | 136506836 | C | T | Het | missense | NOTCH1 | 0.8639782 | . | p.Gly1261Ser | c.3781G>A |
|  | ***chr 12** | **111341933** | **A** | **G** | **Het** | **missense** | **CUX2** | **0.8801122** | **.** | **p.Lys1180Arg** | **c.3539A>G** |
|  | chr17 | 3573721 | T | G | Het | missense | TRPV1 | 0.77420676 | . | p.Tyr683Ser | c.2048A>C |
|  | ***chr 12** | **40340373** | **G** | **A** | **Het** | **missense** | **LRRK2** | **0.7651409** | **.** | **p.Ala2010Thr** | **c.6028G>A** |
|  | chr16 | 2496224 | G | A | Het | missense | TBC1D24 | 0.9306199 | rs369120050 | p.Glu26Lys | c.76G>A |
| Patient 21 | chr9 | 136508295 | C | T | Het | missense | NOTCH1 | 0.8516935 | rs531420022 | p.Gly1088Ser | c.3262G>A |
|  | chr2 | 165326855 | A | G | Het | missense | SCN2A | 0.8016278 | rs1360867886 | p.Thr674Ala | c.2020A>G |
|  | chr11 | 108331945 | G | A | Het | missense | ATM | 0.8967231 | rs1060501604 | p.Ala2566Thr | c.7696G>A |
|  | chr14 | 53951979 | A | G | Het | missense | BMP4 | 0.7866503 | rs1337000514 | p.Tyr82His | c.244T>C |
|  | chr17 | 8576653 | A | G | Het | missense | MYH10 | 0.7731039 | . | p.Val218Ala | c.653T>C |
|  | chr9 | 34234314 | T | G | Het | missense | UBAP1 | 0.95148134 | rs905132769 | p.Cys109Gly | c.325T>G |
| Patient 22 | chr19 | 38506367 | G | T | Het | missense | RYR1 | 0.77305514 | . | p.Arg2869Leu | c.8606G>T |
|  | chr14 | 63981078 | C | A | Het | missense | SYNE2 | 0.7578544 | . | p.Leu581Ile | c.1741C>A |
|  |  |  |  |  |  |  |  |  |  |  |  |
|  | chr21 | 28943686 | C | G | Het | missenset | LTN1 | 0.95617145 | rs376034413 | p.Val1447Leu | c.4339G>C |
|  | chr17 | 43529547 | C | T | Het | Stop gained | ETV4 | 0.888858 | rs1002680213 | p.Trp362* | c.1085G>A |
|  | chr14 | 103673185 | C | T | Het | Splice region variant | KLC1 | 0.81496775 | rs1490837127 | p.Leu559Leu | c.1675C>T |
|  | chr14 | 54902336 | G | C | Het | missense | GCH1 | 0.9336671 | rs748944982 | p.Gln110Glu | c.328C>G |
| Patient 23 | chr17 | 27605482 | G | A | Het | missense | KSR1 | 0.7795605 | rs759287309 | p.Glu500Lys | c.1498G>A |
|  | chr12 | 15982427 | A | G | Het | missense | DERA | 0.76495665 | rs764326230 | p.Met210Val | c.628A>G |
|  | ****chr2** | **211386999** | **C** | **T** | **Het** | **missense** | **ERBB4** | **0.9981347** | **rs770460785** | **p.Arg1112His** | **c.3335G>A** |
|  | chr16 | 89548095 | G | A | Het | missense | SPG7 | 0.9802458 | rs147706568 | p.Val549Met | c.1645G>A |
|  | chr17 | 10447186 | T | C | Het | missense | MYH4 | 0.7575727 | . | p.Arg1666Gly | c.4996A>G |
|  | ***chr1** | **12009614** | **C** | **T** | **Het** | **missense** | **MFN2** | **0.95164794** | **.** | **p.His698Tyr** | **c.2092C>T** |
| Patient 24 | chr14 | 96315439 | T | C | Het | missense | ATG2B | 0.7782087 | . | p.Asn1169Ser | c.3506A>G |
|  | chr1 | 150966808 | T | C | Het | missense | CERS2 | 0.7543513 | rs369491663 | p.Ile266Val | c.796A>G |
|  | ***chr1** | **12009614** | **C** | **T** | **Het** | **missense** | **MFN2** | **0.77919215** | **.** | **p.His698Tyr** | **c.2092C>T** |
|  | chr3 | 49655863 | G | T | Het | missense | BSN | 0.7686863 | rs374754262 | p.Ala2103Ser | c.6307G>T |
|  | chr17 | 10345252 | A | G | Het | missense | MYH13 | 0.7549576 | rs774016181 | p.Phe512Leu | c.1534T>C |
|  | chr12 | 22201593 | G | C | Het | missense | ST8SIA1 | 0.7825114 | . | p.Leu344Val | c.1030C>G |
| Patient 25 | chr19 | 45488511 | A | G | Het | missense | RTN2 | 0.7710584 | rs1010288477 | p.Ile486Thr | c.1457T>C |
|  | chr3 | 1227930 | C | T | Het | Stop gained | CNTN6 | 0.76631606 | . | p.Gln99* | c.295C>T |
|  | chr11 | 46879150 | C | T | Het | missense | LRP4 | 0.87590694 | rs370091369 | p.Val994Ile | c.2980G>A |
|  | chr2 | 8747937 | A | C | Het | missense | KIDINS220 | 0.8575241 | . | p.Ser1160Ala | c.3478T>G |
|  | chr6 | 33264043 | T | G | Het | missense | VPS52 | 0.779939 | rs1210029061 | p.Asn529His | c.1585A>C |
|  | chr5 | 251460 | G | C | Het | missense | SDHA | 0.972038 | . | p.Asp596His | c.1786G>C |
| Patient 26 | chr7 | 27785260 | A | T | Het | missense | TAX1BP1 | 0.7585856 | rs1200240156 | p.Asp237Val | c.710A>T |
|  | chr6 | 22292639 | G | A | Het | missense | PRL | 0.7612938 | rs201458155 | p.Arg72Trp | c.214C>T |
|  | chr15 | 42439744 | T | C | Het | missense | ZNF106 | 0.95821786 | . | p.Glu1255Gly | c.3764A>G |
|  | chr5 | 135343416 | T | C | Het | missense | MACROH2A1 | 0.7596569 | rs770424510 | p.His266Arg | c.797A>G |
|  | chr3 | 69959391 | G | A | Het | missense | MITF | 0.7581221 | rs202020443 | p.Ala384Thr | c.1150G>A |
|  | chr20 | 64213637 | C | T | Het | missense | MYT1 | 0.77001196 | rs776604003 | p.Arg541Trp | c.1621C>T |
| Patient 27 | chr12 | 54404795 | A | C | Het | missense | ITGA5 | 0.7551581 | . | p.Val442Gly | c.1325T>G |
|  | chr8 | 23433037 | C | CA | Het | Frameshift elongation | ENTPD4 | 0.7549797 | . | p.Tyr581fs | c.1739dupT |
|  | chr17 | 9023008 | C | T | Het | missense | NTN1 | 0.7827309 | . | p.Pro212Leu | c.635C>T |
|  | chr8 | 17373150 | C | G | Het | missense | MTMR7 | 0.78149843 | rs779144225 | p.Glu39Gln | c.115G>C |
|  | chr19 | 58212300 | TG | T | Het | Frameshift truncation | ZNF274 | 0.757672 | . | p.Val375fs | c.1123delG |
|  | chr19 | 1054633 | G | A | Het | missense | ABCA7 | 0.997047 | rs771155690 | p.Gly1264Arg | c.3790G>A |
| Patient 28 | chr4 | 6301924 | C | G | Het | missense | WFS1 | 0.97929305 | rs200136995 | p.Thr710Ser | c.2129C>G |
|  | chr2 | 178072078 | CTCTT | C | Het | Frameshift truncation | PDE11A | 0.95166695 | . | p.Lys119fs | c.356359delAAGA |
|  | chr8 | 125067692 | C | T | Het | missense | WASHC5 | 0.7874825 | rs151298198 | p.Arg393His | c.1178G>A |
|  | chr5 | 97171210 | C | A | Het | Stop gained | RIOK2 | 0.76378715 | . | p.Glu259* | c.775G>T |
|  | chr22 | 42212034 | T | C | Het | missense | TCF20 | 0.7541088 | . | p.Gln1091Arg | c.3272A>G |
|  | chr19 | 10140257 | C | T | Het | missense | DNMT1 | 0.9796639 | rs780717834 | p.Gly1199Ser | c.3595G>A |
| Patient 29 | chr15 | 33603124 | A | G | Het | missense | RYR3 | 0.7688583 | rs146818373 | p.Ile642Val | c.1924A>G |
|  | chr2 | 219290270 | C | T | Het | missense | PTPRN | 0.785356 | rs139964061 | p.Val966Met | c.2896G>A |
|  | chr17 | 8484215 | C | G | Het | missense | MYH10 | 0.7731039 | . | p.Asp1700His | c.5098G>C |
|  | chr8 | 468793 | T | C | Het | missense | FBXO25 | 0.75425696 | rs756280057 | p.Phe365Leu | c.1093T>C |
|  | chr11 | 128480407 | C | T | Het | missense | ETS1 | 0.7532589 | . | p.Asp303Asn | c.907G>A |
|  | ***chr 15** | **89325456** | **G** | **C** | **Het** | **missense** | **POLG** | **0.9798247** | **rs796052906** | **p.Pro648Arg** | **c.1943C>G** |
| Patient 30 | chr11 | 17387424 | G | C | Het | missense | KCNJ11 | 0.9352639 | rs561086953 | p.Thr223Ser | c.668C>G |
|  | ***chr9** | **113260405** | **G** | **A** | **Het** | **missense** | **SLC31A1** | **0.7846043** | **rs538420854** | **p.Gly169Ser** | **c.505G>A** |
|  | chr4 | 169477204 | C | T | Het | missense | NEK1 | 0.99424475 | rs757319297 | p.Arg785His | c.2354G>A |
|  | ***chr 15** | **89325456** | **G** | **C** | **Het** | **missense** | **POLG** | **0.78877246** | **rs796052906** | **p.Pro648Arg** | **c.1943C>G** |
|  | ***chr9** | **113260405** | **G** | **A** | **Het** | **missense** | **SLC31A1** | **0.7775436** | **rs538420854** | **p.Gly169Ser** | **c.505G>A** |
|  | chr6 | 170572265 | A | G | Het | Stop lost | TBP | 0.9795824 | rs756475719 | p.Ter340Ter | c.1020A>G |
| Patient 31 | chr1 | 160127617 | G | A | Het | missense | ATP1A2 | 0.95033735 | rs369898494 | p.Ala272Thr | c.814G>A |
|  | chr6 | 32581773 | A | C | Het | missense | HLA-DRB1 | 0.8237776 | . | p.Cys146Gly | c.436T>G |
|  | chr21 | 44312234 | A | T | Het | missense | PFKL | 0.82649827 | rs930410431 | p.Thr123Ser | c.367A>T |
|  | chr10 | 100329270 | A | T | Het | missense | PKD2L1 | 0.768237 | . | p.Ile97Asn | c.290T>A |
|  | chr18 | 8784508 | C | T | Het | missense | MTCL1 | 0.7613257 | rs115973534 | p.Arg826Trp | c.2476C>T |
| Patient 32 | chr2 | 177663894 | A | G | Het | missense | PDE11A | 0.9506036 | rs371810618 | p.Ile873Thr | c.2618T>C |
|  | chr11 | 17387287 | C | T | Het | missense | KCNJ11 | 0.93254524 | rs756552714 | p.Asp269Asn | c.805G>A |
|  | chr16 | 71745604 | G | A | Het | missense | AP1G1 | 0.77937186 | rs565343527 | p.Leu584Phe | c.1750C>T |
|  | ****chr14** | **20693932** | **G** | **C** | **Het** | **missense** | **ANG** | **0.99499243** | **rs535311762** | **p.Gly123Ala** | **c.368G>C** |
|  | chr17 | 27778920 | G | A | Het | missense | NOS2 | 0.909904 | rs377644483 | p.Arg381Trp | c.1141C>T |
|  | chr11 | 108331945 | G | A | Het | missense | ATM | 0.8967231 | rs1060501604 | p.Ala2566Thr | c.7696G>A |
| Patient 33 | chr19 | 1058213 | G | A | Het | missense | ABCA7 | 0.9968668 | rs144259338 | p.Arg1698Gln | c.5093G>A |
|  | chr2 | 165092430 | A | G | Het | missense | SCN3A | 0.9510041 | rs751272471 | p.Met1544Thr | c.4631T>C |
|  | chr20 | 38728508 | C | T | Het | missense | SLC32A1 | 0.7692064 | rs547270146 | p.Arg483Cys | c.1447C>T |
|  | chr11 | 101962713 | A | C | Het | missense | CEP126 | 0.9862319 | . | p.Lys560Gln | c.1678A>C |
|  | chr15 | 67066246 | G | C | Het | missense | SMAD3 | 0.9308975 | rs1443339510 | p.Cys31Ser | c.92G>C |
|  | chr15 | 75356853 | G | A | Het | missense | MAN2C1 | 0.91130775 | rs377575239 | p.Ala883Val | c.2648C>T |
| Patient 34 | chr11 | 118504857 | G | A | Het | missense | KMT2A | 0.93032956 | rs143843795 | p.Glu2989Lys | c.8965G>A |
|  | chr12 | 47751928 | C | G | Het | missense | RAPGEF3 | 0.77036095 | . | p.Glu87Asp | c.261G>C |
|  | chr9 | 133070501 | C | T | Het | missense | CEL | 0.7681166 | rs1316968646 | p.Arg446Trp | c.1336C>T |
|  | ****chr9** | **132330484** | **T** | **G** | **Het** | **missense** | **SETX** | **0.9951079** | **rs145145045** | **p.Thr372Pro** | **c.1114A>C** |
|  | chr16 | 89553844 | A | T | Het | Stop gained | SPG7 | 0.98079085 | . | p.Lys663* | c.1987A>T |
|  | chr21 | 44325985 | C | T | Het | missense | PFKL | 0.826109 | . | p.Arg672Trp | c.2014C>T |
| Patient 35 | chr7 | 596277 | T | C | Het | missense | PRKAR1B | 0.9966212 | . | p.Asn193Asp | c.577A>G |
|  | chr16 | 78386906 | G | T | Het | missense | WWOX | 0.95166695 | . | p.Arg188Leu | c.563G>T |
|  | chr1 | 149944207 | C | T | Het | missense | OTUD7B | 0.8284529 | rs201225347 | p.Ala728Thr | c.2182G>A |
|  | chr18 | 163448 | C | A | Het | missense | USP14 | 0.82525384 | . | p.Leu53Ile | c.157C>A |
|  | chr17 | 68430114 | A | G | Het | missense | WIPI1 | 0.7717196 | rs191306252 | p.Phe283Leu | c.847T>C |
|  | chr9 | 134727269 | G | A | Het | missense | COL5A1 | 0.76439553 | . | p.Asp220Asn | c.658G>A |
| Patient 36 | ***chr5** | **179836586** | **C** | **T** | **Het** | **missense** | **SQSTM1** | **0.9979669** | **rs199854262** | **p.Pro439Leu** | **c.1316C>T** |
|  | chr19 | 13235003 | C | T | Het | missense | CACNA1A | 0.8822619 | rs931067957 | p.Glu1729Lys | c.5185G>A |
|  | chr2 | 166073480 | C | T | Het | missense | SCN1A | 0.9498747 | . | p.Gly48Ser | c.142G>A |
|  | ***chr5** | **179836586** | **C** | **T** | **Het** | **missense** | **SQSTM1** | **0.99416006** | **rs199854262** | **p.Pro439Leu** | **c.1316C>T** |
|  | chr8 | 103885602 | A | C | Het | missense | RIMS2 | 0.77190316 | . | p.Ser113Arg | c.337A>C |
|  | chr12 | 124795223 | G | T | Het | missense | SCARB1 | 0.75836986 | rs755969350 | p.Leu392Ile | c.1174C>A |
| Patient 37 | chr7 | 117540230 | C | T | Het | missense | CFTR | 0.7635801 | rs121909011 | p.Arg334Trp | c.1000C>T |
|  | chr5 | 58455339 | A | T | Het | missense | PLK2 | 0.76048523 | . | p.Ser567Arg | c.1701T>A |
|  | chr5 | 168431437 | A | C | Het | missense | WWC1 | 0.7560892 | . | p.Glu758Ala | c.2273A>C |
|  | chr17 | 4946003 | G | A | Het | Splice region variant | PFN1 | 0.98848516 | rs778846806 | . | c.*987G>A |
|  | chr3 | 184165426 | C | T | Het | missense | DVL3 | 0.8045458 | rs763604484 | p.Ser233Leu | c.698C>T |
|  | chr3 | 184170113 | T | C | Het | missense | DVL3 | 0.8045458 | rs1018717196 | p.Phe536Leu | c.1606T>C |
| Patient 38 | chr19 | 53893008 | A | G | Het | missense | PRKCG | 0.95166695 | . | p.Glu281Gly | c.842A>G |
|  | chr19 | 15650023 | G | C | Het | missense | CYP4F3 | 0.7558957 | rs750565828 | p.Gly253Ala | c.758G>C |
|  | chr10 | 94942294 | T | C | Het | missense | CYP2C9 | 0.7567127 | . | p.Val145Ala | c.434T>C |
|  | chr21 | 44313961 | C | A | Het | missense | PFKL | 0.8259968 | rs761447739 | p.Phe229Leu | c.687C>A |
|  | chr1 | 154965967 | G | A | Het | missense | SHC1 | 0.8219971 | . | p.Pro456Ser | c.1366C>T |
|  | chr22 | 38221699 | G | T | Het | missense | TMEM184B | 0.7830759 | rs958897396 | p.Pro332Thr | c.994C>A |
| Patient 39 | ***chr2** | **232819840** | **G** | **A** | **Het** | **missense** | **GIGYF2** | **0.97861487** | **rs200601366** | **p.Arg817His** | **c.2450G>A** |
|  | chr2 | 73641562 | G | A | Het | missense | NAT8 | 0.7875869 | rs555774069 | p.Arg23Trp | c.67C>T |
|  | chr17 | 5377164 | C | T | Het | missense | RABEP1 | 0.76694286 | rs779665772 | p.Arg692Trp | c.2074C>T |
|  | ***chr2** | **232819840** | **G** | **A** | **Het** | **missense** | **GIGYF2** | **0.9842669** | **rs200601366** | **p.Arg817His** | **c.2450G>A** |
|  | chr2 | 32597922 | C | G | Het | missense | BIRC6 | 0.76502943 | rs1361534341 | p.Ser4595Cys | c.13784C>G |
|  | chr6 | 36684200 | C | G | Het | missense | CDKN1A | 0.76255167 | rs765503766 | p.Asp33Glu | c.99C>G |
| Patient 40 | ***chr 12** | **79296116** | **G** | **C** | **Het** | **missense** | **SYT1** | **0.7773408** | **.** | **p.Met174Ile** | **c.522G>C** |
|  | ***chr1** | **22576340** | **C** | **G** | **Het** | **missense** | **EPHA8** | **0.7598074** | **.** | **p.Arg95Gly** | **c.283C>G** |
|  | chr15 | 92974874 | T | G | Het | Splice region | CHD2 | 0.758292 | . | . | c.2506-5T>G |
|  | chr8 | 104248736 | G | A | Het | missense | RIMS2 | 0.7710175 | rs894767821 | p.Ser1189Asn | c.3566G>A |
|  | ***chr 12** | **79296116** | **G** | **C** | **Het** | **missense** | **SYT1** | **0.76878065** | **.** | **p.Met174Ile** | **c.522G>C** |
|  | ***chr1** | **22576340** | **C** | **G** | **Het** | **missense** | **EPHA8** | **0.7565755** | **.** | **p.Arg95Gly** | **c.283C>G** |
| Patient 41 | chr6 | 158770844 | T | C | Het | missense | EZR | 0.81764364 | rs1056974 | p.Lys337Arg | c.1010A>G |
|  | chr10 | 87965443 | G | C | Het | missense | PTEN | 0.7623655 | . | p.Asp395His | c.1183G>C |
|  | chr19 | 38511586 | G | A | Het | missense | RYR1 | 0.7610434 | rs200797340 | p.Val3050Ile | c.9148G>A |
|  | ****chr3** | **52696595** | **GT** | **G** | **Het** | **Frameshift truncation** | **GLT8D1** | **0.9946762** | **rs1308367710** | **p.Lys131fs** | **c.393delA** |
|  | chr2 | 63914222 | A | G | Het | missense | VPS54 | 0.984076 | rs1305538269 | p.Val765Ala | c.2294T>C |
|  | chr16 | 89544642 | C | T | Het | Splice region variant | SPG7 | 0.88822854 | rs371986686 |  | c.1325-6C>T |
| Patient 42 | chr11 | 17772595 | G | C | Het | missense | KCNC1 | 0.95148593 | rs1286132223 | p.Ala501Pro | c.1501G>C |
|  | chr22 | 36237085 | C | T | Het | missense | APOL2 | 0.93354034 | rs754005748 | p.Cys60Tyr | c.179G>A |
|  | chr19 | 32711760 | C | G | Het | missense | NUDT19 | 0.78409004 | rs766234535 | p.Leu311Val | c.931C>G |
|  | chr11 | 101962713 | A | C | Het | missense | CEP126 | 0.9862319 | . | p.Lys560Gln | c.1678A>C |
|  | chr18 | 724504 | T | C | Het | missense | YES1 | 0.7585862 | . | p.Thr523Ala | c.1567A>G |
|  | chr6 | 38938859 | G | A | Het | missense | DNAH8 | 0.75611514 | rs940769485 | p.Glu3960Lys | c.11878G>A |
| Patient 43 | chr2 | 219214460 | G | A | Het | missense | ABCB6 | 0.76850283 | rs746772305 | p.Arg439Cys | c.1315C>T |
|  | ***chr2** | **221426049** | **G** | **C** | **Het** | **missense** | **EPHA4** | **0.7604259** | **rs373468133** | **p.His980Gln** | **c.2940C>G** |
|  | chr9 | 5080268 | T | C | Het | missense | JAK2 | 0.76021385 | rs372254348 | p.Ile724Thr | c.2171T>C |
|  | ***chr2** | **221426049** | **G** | **C** | **Het** | **missense** | **EPHA4** | **0.9946709** | **rs373468133** | **p.His980Gln** | **c.2940C>G** |
|  | chr2 | 21025070 | ATT | A | Het | Frameshift truncation | APOB | 0.77995723 | . | p.Lys766fs | c.2297_2298delAA |
|  | chr6 | 75130099 | C | G | Het | missense | COL12A1 | 0.763051 | . | p.Asp2068His | c.6202G>C |
| Patient 44 | chr12 | 120988859 | T | C | Het | missense | HNF1A | 0.9357458 | . | p.Met118Thr | c.353T>C |
|  | chr1 | 46032614 | C | A | Het | missense | MAST2 | 0.7909005 | . | p.Pro1145Thr | c.3433C>A |
|  | chr19 | 18177220 | G | T | Het | missense | IFI30 | 0.8225083 | rs1263684547 | p.Met188Ile | c.564G>T |
|  | chr4 | 95125075 | T | A | Het | missense | BMPR1B | 0.7930536 | rs776445882 | p.Ile210Asn | c.629T>A |
|  | chr6 | 149717968 | C | CG | Het | Frameshift elongation | LATS1 | 0.7640141 | rs776480344 | p.Leu96fs | c.285dupG |
|  | chr20 | 62822601 | G | A | Het | missense | COL9A3 | 0.76356274 | rs1159923081 | p.Gly163Glu | c.488G>A |
| Patient 45 | chr2 | 73641562 | G | A | Het | missense | NAT8 | 0.7875869 | rs555774069 | p.Arg23Trp | c.67C>T |
|  | chr6 | 20955421 | G | A | Het | missense | CDKAL1 | 0.7839614 | rs975051721 | p.Gly249Ser | c.745G>A |
|  | chr11 | 959457 | A | G | Het | missense | AP2A2 | 0.76621574 | rs200802126 | p.Ile30Val | c.88A>G |
|  | chr2 | 120989932 | G | C | Het | missense | GLI2 | 0.95492315 | rs759453086 | p.Val1340Leu | c.4018G>C |
|  | chr17 | 1469570 | C | G | Het | missense | MYO1C | 0.777476 | rs777514075 | p.Trp857Cys | c.2571G>C |
|  | chr1 | 22576620 | T | G | Het | missense | EPHA8 | 0.7570623 | . | p.Ile188Arg | c.563T>G |
| Patient 46 | chr10 | 101810270 | C | G | Het | missense | OGA | 0.7672779 | rs766002461 | p.Glu132Gln | c.394G>C |
|  | chr13 | 91693709 | C | T | Het | missense | GPC5 | 0.7566766 | rs145336505 | p.Ala283Val | c.848C>T |
|  | ***chr4** | **1802954** | **G** | **A** | **Het** | **missense** | **FGFR3** | **0.75714046** | **rs750429622** | **p.Arg324His** | **c.971G>A** |
|  | chr7 | 83392585 | C | T | Het | missense | SEMA3E | 0.95283294 | rs769248913 | p.Arg546Gln | c.1637G>A |
|  | chr2 | 8827071 | C | T | Het | missense | KIDINS220 | 0.85563445 | rs202051983 | p.Ser8Asn | c.23G>A |
|  | ***chr4** | **1802954** | **G** | **A** | **Het** | **missense** | **FGFR3** | **0.81086326** | **rs750429622** | **p.Arg324His** | **c.971G>A** |
| Patient 47 | chr1 | 179846391 | G | A | Het | missense | TOR1AIP2 | 0.86678505 | rs1183676207 | p.His365Tyr | c.1093C>T |
|  | chr2 | 189564079 | A | T | Het | missense | SLC40A1 | 0.76942956 | . | p.Tyr303Asn | c.907T>A |
|  | ***chr 22** | **36300145** | **C** | **G** | **Het** | **missense** | **MYH9** | **0.76301277** | **rs199520053** | **p.Gln986His** | **c.2958G>C** |
|  | ***chr 22** | **36300145** | **C** | **G** | **Het** | **missense** | **MYH9** | **0.7681934** | **rs199520053** | **p.Gln986His** | **c.2958G>C** |
|  | chr11 | 66285738 | C | T | Het | missense | YIF1A | 0.75964254 | rs747449469 | p.Val150Met | c.448G>A |
|  | chr9 | 128822522 | G | A | Het | missense | ENDOG | 0.7592865 | rs777639024 | p.Ser269Asn | c.806G>A |
| Patient 48 | chr15 | 93000536 | C | T | Het | missense | CHD2 | 0.9514792 | rs1273391509 | p.Arg1345Trp | c.4033C>T |
|  | chr1 | 160166556 | T | C | Het | missense | ATP1A4 | 0.7694338 | rs898416231 | p.Met359Thr | c.1076T>C |
|  | ***chr 10** | **100296184** | **C** | **T** | **Het** | **missense** | **PKD2L1** | **0.76118016** | **rs575825254** | **p.Ala432Thr** | **c.1294G>A** |
|  | chr15 | 42449798 | T | C | Het | missense | ZNF106 | 0.96065676 | rs773509399 | p.Lys802Arg | c.2405A>G |
|  | chr11 | 46898659 | C | T | Het | missense | LRP4 | 0.87271005 | rs370407081 | p.Arg232His | c.695G>A |
|  | ***chr 10** | **100296184** | **C** | **T** | **Het** | **missense** | **PKD2L1** | **0.7653965** | **rs575825254** | **p.Ala432Thr** | **c.1294G>A** |
| Patient 49 | chr11 | 35317443 | G | A | Het | missense | SLC1A2 | 0.95013183 | rs750357702 | p.Arg31Trp | c.91C>T |
|  | chr6 | 52273285 | C | A | Het | missense | MCM3 | 0.7539389 | . | p.Asp586Tyr | c.1756G>T |
|  | chr17 | 19283349 | C | T | Het | missense | EPN2 | 0.752958 | . | p.Ala77Val | c.230C>T |
|  | chr8 | 138821402 | G | A | Het | missense | COL22A1 | 0.7613015 | rs371733795 | p.Arg327Trp | c.979C>T |
|  | chr2 | 237379158 | G | A | Het | missense | COL6A3 | 0.75515467 | rs146291186 | p.Arg659Cys | c.1975C>T |
|  | chr13 | 20821710 | T | G | Het | missense | XPO4 | 0.7535537 | rs905963387 | p.Glu389Asp | c.1167A>C |
| Patient 50 | chr12 | 12121168 | G | C | Het | missense | LRP6 | 0.78840035 | rs138902458 | p.His1600Gln | c.4800C>G |
|  | ***chr3** | **142748331** | **A** | **G** | **Het** | **missense** | **TRPC1** | **0.76972204** | **rs759725115** | **p.His168Arg** | **c.503A>G** |
|  | chr17 | 7286520 | G | A | Het | missense | SLC2A4 | 0.7779792 | rs70937028 | p.Arg474Gln | c.1421G>A |
|  | ***chr3** | **142748331** | **A** | **G** | **Het** | **missense** | **TRPC1** | **0.77493733** | **rs759725115** | **p.His168Arg** | **c.503A>G** |
|  | chr5 | 133086837 | C | T | Het | missense | HSPA4 | 0.7586539 | rs368924343 | p.Arg322Cys | c.964C>T |
|  | chr1 | 1628321 | G | C | Het | missense | MIB2 | 0.75578606 | . | p.Lys745Asn | c.2235G>C |
| **Patient 51** | ****chr2** | **74370248** | **G** | **A** | **Het** | **missense** | **DCTN1** | **0.9980531** | **rs150368544** | **p.Arg409Trp** | **c.1225C>T** |
|  | chr17 | 3615387 | T | C | Het | missense | SHPK | 0.8788706 | rs186122425 | p.Asn325Ser | c.974A>G |
|  | chr14 | 24608374 | G | A | Het | missense | GZMH | 0.77741563 | rs751938436 | p.Arg32Cys | c.94C>T |
|  | ****chr2** | **74370248** | **G** | **A** | **Het** | **missense** | **DCTN1** | **0.99440664** | **rs150368544** | **p.Arg409Trp** | **c.1225C>T** |
|  | chr11 | 20160270 | G | A | Het | missense | DBX1 | 0.860489 | . | p.Pro19Ser | c.55C>T |
|  | chr2 | 151883331 | C | T | Het | missense | CACNB4 | 0.8528925 | rs886054969 | p.Asp63Asn | c.187G>A |
| Patient 52 | ***chr 11** | **121566942** | **A** | **G** | **Het** | **missense** | **SORL1** | **0.9971217** | **.** | **p.Ser1018Gly** | **c.3052A>G** |
|  | chr2 | 165130164 | C | T | Het | missense | SCN3A | 0.95135814 | rs749586926 | p.Gly900Ser | c.2698G>A |
|  | chr2 | 165389318 | C | G | Het | missense | SCN2A | 0.80303127 | . | p.Gln1838Glu | c.5512C>G |
|  | ***chr 11** | **121566942** | **A** | **G** | **Het** | **missense** | **SORL1** | **0.87699646** | **.** | **p.Ser1018Gly** | **c.3052A>G** |
|  | chr6 | 111702895 | T | G | Het | missense | FYN | 0.76389605 | rs77439992 | p.Gln229His | c.687A>C |
|  | chr6 | 149676632 | C | T | Het | missense | LATS1 | 0.75986564 | rs575740406 | p.Arg900His | c.2699G>A |
| Patient 53 | chr17 | 17814654 | G | A | Het | missense | SREBF1 | 0.79024523 | rs775182965 | p.Pro929Leu | c.2786C>T |
|  | chr9 | 128389375 | G | C | Het | missense | URM1 | 0.76708025 | . | p.Trp101Cys | c.303G>C |
|  | chr12 | 6341799 | C | T | Het | missense | TNFRSF1A | 0.7555227 | rs772424047 | p.Val6Met | c.16G>A |
|  | chr12 | 98727323 | G | A | Het | Splice region variant | APAF1 | 0.9614157 | rs758448810 | . | c.3600+7G>A |
|  | chr17 | 27762908 | A | G | Het | missense | NOS2 | 0.9136475 | . | p.Leu897Pro | c.2690T>C |
|  | chr2 | 8731201 | G | T | Het | missense | KIDINS220 | 0.8500276 | rs201737651 | p.Ala1612Glu | c.4835C>A |
|  | chr6 | 20781168 | C | T | Het | Stop gained | CDKAL1 | 0.7839614 | . | p.Gln181* | c.541C>T |
| Patient 54 | chr1 | 96770729 | G | A | Het | missense | PTBP2 | 0.7762759 | rs777662925 | p.Glu104Lys | c.310G>A |
|  | ***chr3** | **183833764** | **G** | **C** | **Het** | **missense** | **PARL** | **0.7618882** | **rs554169588** | **p.Ala297Gly** | **c.890C>G** |
|  | chr12 | 98708697 | G | T | Het | missense | APAF1 | 0.99038625 | rs146420669 | p.Arg945Leu | c.2834G>T |
|  | chr22 | 37983605 | G | C | Het | missense | SOX10 | 0.9601007 | . | p.Asp60Glu | c.180C>G |
|  | ***chr3** | **183833764** | **G** | **C** | **Het** | **missense** | **PARL** | **0.79481125** | **rs554169588** | **p.Ala297Gly** | **c.890C>G** |
| Patient 55 | chr8 | 52683691 | T | A | Het | missense | RB1CC1 | 0.792855 | . | p.Lys76Ile | c.227A>T |
|  | chr17 | 17817344 | C | G | Het | missense | SREBF1 | 0.78863674 | rs367758777 | p.Leu536Phe | c.1608G>C |
|  | chr12 | 112938630 | GGC | G | Het | Frameshift truncation | OAS3 | 0.75428236 | rs768202617 | p.Ala35fs | c.103104delGC |
|  | chr12 | 56093538 | C | T | Het | missense | ERBB3 | 0.9624983 | rs775042280 | p.Arg490Cys | c.1468C>T |
|  | chr12 | 39370200 | G | A | Het | missense | KIF21A | 0.85042065 | rs200140335 | p.Pro36Ser | c.106C>T |
|  | chr11 | 66423858 | G | A | Het | missense | NPAS4 | 0.8408511 | rs751922807 | p.Arg323His | c.968G>A |
| Patient 56 | chr20 | 58853383 | G | A | Het | missense | GNAS | 0.9486675 | rs776193516 | p.Gly40Ser | c.118G>A |
|  | chr22 | 41884872 | A | T | Het | missense | SREBF2 | 0.79152167 | . | p.Asp690Val | c.2069A>T |
|  | chr17 | 27588511 | A | G | Het | missense | KSR1 | 0.782334 | rs1409707417 | p.Asp264Gly | c.791A>G |
|  | chr16 | 2455400 | G | A | Het | missense | CCNF | 0.99416786 | rs369730776 | p.Arg574Gln | c.1721G>A |
|  | chr13 | 77083105 | G | A | Het | missense | MYCBP2 | 0.86533946 | rs777629569 | p.Arg3617Cys | c.10849C>T |
|  | chr11 | 20157198 | G | T | Het | missense | DBX1 | 0.85580057 | rs200639137 | p.Pro171Thr | c.511C>A |
| Patient 57 | chr 1 | 212066870 | C | G | Het | missense | DTL | 0.7664086 | rs915464529 | p.Ala233Gly | c.698C>G |
|  | chr16 | 68364875 | G | C | Het | missense | SMPD3 | 0.7603931 | rs907687973 | p.Asp477Glu | c.1431C>G |
|  | chr16 | 68365074 | G | C | Het | missense | SMPD3 | 0.7603931 | rs542551484 | p.Pro448Ala | c.1342C>G |
|  | ****chr16** | **2437155** | **G** | **A** | **Het** | **missense** | **CCNF** | **0.99468136** | **.** | **p.Glu125Lys** | **c.373G>A** |
|  | chr10 | 43112107 | G | A | Het | missense | RET | 0.86919147 | rs201553718 | p.Glu511Lys | c.1531G>A |
|  | chr16 | 2472218 | C | T | Het | missense | NTN3 | 0.76051795 | rs919440296 | p.Pro173Ser | c.517C>T |
|  |  |  |  |  |  |  |  |  |  |  |  |

* indicates overlap with the FTD or ALS variants from Exomiser; ** indicates overlap with interesting variants in Table 1. FTD variants are highlighted in white and ALS variants are highlighted in gray. Chr: chromosome; Pos: genomic position using reference hg38; Ref: reference allele; Alt: alternative allele; Rsid: dbSNP reference SNP number.

|  |  |  |  |  |  |  |  |  |  |  |  |
| --- | --- | --- | --- | --- | --- | --- | --- | --- | --- | --- | --- |
